# Development of Wheat-*Aegilops caudata* Introgression Lines and Their Characterization Using Genome-Specific KASP Markers

**DOI:** 10.3389/fpls.2020.00606

**Published:** 2020-05-14

**Authors:** Surbhi Grewal, Manel Othmeni, Jack Walker, Stella Hubbart-Edwards, Cai-yun Yang, Duncan Scholefield, Stephen Ashling, Peter Isaac, Ian P. King, Julie King

**Affiliations:** ^1^Division of Plant and Cop Sciences, Nottingham BBSRC Wheat Research Centre, University of Nottingham, Nottingham, United Kingdom; ^2^IDna Genetics Ltd., Norwich Research Park, Norwich, United Kingdom

**Keywords:** wheat, *Aegilops caudata*, introgression, recombinant chromosomes, KASP, GISH

## Abstract

*Aegilops caudata* L. [syn. *Ae. markgrafii* (Greuter) Hammer], is a diploid wild relative of wheat (2n = 2x = 14, CC) and a valuable source for new genetic diversity for wheat improvement. It has a variety of disease resistance factors along with tolerance for various abiotic stresses and can be used for wheat improvement through the generation of genome-wide introgressions resulting in different wheat–*Ae. caudata* recombinant lines. Here, we report the generation of nine such wheat–*Ae. caudata* recombinant lines which were characterized using wheat genome-specific KASP (Kompetitive Allele Specific PCR) markers and multi-color genomic *in situ* hybridization (mcGISH). Of these, six lines have stable homozygous introgressions from *Ae. caudata* and will be used for future trait analysis. Using cytological techniques and molecular marker analysis of the recombinant lines, 182 KASP markers were physically mapped onto the seven *Ae. caudata* chromosomes, of which 155 were polymorphic specifically with only one wheat subgenome. Comparative analysis of the physical positions of these markers in the *Ae. caudata* and wheat genomes confirmed that the former had chromosomal rearrangements with respect to wheat, as previously reported. These wheat–*Ae. caudata* recombinant lines and KASP markers are useful resources that can be used in breeding programs worldwide for wheat improvement. Additionally, the genome-specific KASP markers could prove to be a valuable tool for the rapid detection and marker-assisted selection of other Aegilops species in a wheat background.

## Introduction

Wheat is one of the most widely cultivated crops worldwide, contributing about a fifth of the total calories and protein consumed by humans. Although wheat breeders report annual wheat yield gains, they are not sufficient to meet the needs of an ever-growing population. This is due to a lack of genetic diversity present in the gene pool of wheat that can be utilized for the development of higher yielding wheat varieties adapted to climate change. However, wild relatives of wheat possess genetic diversity that could be exploited in wheat breeding programs through introgression of small segments of their genome, carrying desirable traits, into wheat (Feuillet et al., 2008).

*Aegilops caudata* L. [syn. *Ae. markgrafii* (Greuter) Hammer] is a diploid wild relative (2n = 2x = 14, CC) of hexaploid wheat, *Triticum aestivum* (2n = 6x = 42, AABBDD). It is native to the north-eastern Mediterranean basin with the main distribution from Greece to northern Iraq (Ohta and Yasukawa, 2015). It contributed the C-genome to allotetraploids *Aegilops cylindrica* (2n = 4x = 28; DDCC) and *Aegilops truncialis* (2n = 4x = 28; UUCC). *Ae. caudata* carries resistance genes for several diseases (Makkouk et al., 1994), especially against stripe rust (Valkoun et al., 1985; Baldauf et al., 1992; Toor et al., 2016), leaf rust (Iqbal et al., 2007; Riar et al., 2012; Gong et al., 2017), stem rust (Dyck et al., 1990; Gong et al., 2017) and powdery mildew (Gill et al., 1985; Valkoun et al., 1985; Baldauf et al., 1992; Weidner et al., 2012) and resistance against pests such as greenbug and hessian fly (Gill et al., 1985; Baldauf et al., 1992). Moreover, some accessions of *Ae. caudata* have shown tolerance to abiotic stresses such as freezing (Barashkova and Migushova, 1984; Iriki et al., 2001) and salinity (Gorham, 1990) and seed quality traits, such as increased grain mineral content (Wang et al., 2011). Thus *Ae. caudata* provides an important source of genetic variation for agronomically important traits that can be transferred into wheat as has been previously done for other Aegilops species (King et al., 2017, 2018).

In the past, *Ae. caudata* was not widely used in wheat pre-breeding programs due to the inability to identify the C-genome chromosomes in a wheat background. In addition, poor knowledge of the C-genome organization and syntenic relationships between wheat and *Ae. caudata* chromosomes has hampered the use of its genetic potential in wheat breeding. Over the past decades, however, efforts have been made to study the molecular organization of the *Ae. caudata genome* and its homology with wheat homoeologous groups. Due to the presence of metacentric and submetacentric chromosomes, *Ae. caudata* has been shown to have a highly asymmetric karyotype (Friebe et al., 1992). This is distinct from most of the Triticeae species and would suggest that chromosome collinearity in *Ae. caudata* is distorted compared to wheat. Fluorescence *in situ* hybridization (FISH) and molecular marker analysis of flow-sorted C-genome chromosomes have also confirmed genome rearrangements in *Ae. caudata* (Molnár et al., 2015, 2016). A set of wheat (cv. Alcedo)-*Ae. caudata* addition lines B-G (Schubert and Blüthner, 1992, 1995) have also been characterized extensively in previous studies using cytogenetic markers (Friebe et al., 1992), isozyme analysis (Schmidt et al., 1993), Simple Sequence Repeat (SSR) markers (Peil et al., 1998; Gong et al., 2017; Niu et al., 2018), FISH with cDNA probes (Danilova et al., 2017), Conserved Orthologous Sequence (COS) and PCR-based Landmark Unique Gene (PLUG) markers (Gong et al., 2017), and sequential FISH and genomic *in situ* hybridization (GISH) (Niu et al., 2018). Even though most of these studies found that the set of addition lines carried chromosomal rearrangements compared to wheat, the assignment of *Ae. caudata* chromosomes to corresponding wheat homoeologous groups was not consistent between them.

Despite numerous studies using wheat-*Ae. caudata* addition lines and the development of molecular markers capable of detecting *Ae. caudata* chromosomes in a wheat background, its utilization for wheat improvement through recombination with wheat chromosomes has rarely been reported (Dyck et al., 1990; Iqbal et al., 2007; Riar et al., 2012; Toor et al., 2016; Weidner et al., 2012). In this work, we report the generation of six stable wheat-*Ae. caudata* recombinant lines and the physical location of 182 KASP markers on *Ae. caudata* chromosomes, that can be used to detect the presence of *Ae. caudata* introgressions in a wheat background.

## Materials and Methods

### Plant Material

Bread wheat cv. Paragon *ph1/ph1* mutant was pollinated with *Ae. caudata* accession 2090001 (obtained from Germplasm Resource Unit, GRU at the John Innes Centre, Norwich, United Kingdom) to produce F_1_ hybrids. The origin of accession 2090001, according to the GRU database Seedstor, is unknown. There is no trait data available for this accession and it was thus, chosen at random. *T. aestivum* cv. Alcedo- *Ae. caudata* disomic addition lines B-G (TA3558-TA3563) carrying *Ae. caudata* chromosomes 2C, 5C, 6C, 7C, 3C, and 4C respectively, and the Alcedo cultivar were obtained from Wheat Genetics Resource Center (WGRC) at Kansas State University, United States.

The crossing program is as described in Grewal et al. (2018). In summary, the F_1_ interspecific hybrids were grown to maturity and used as the female parent while backcrossed with Paragon wheat, *Ph1/Ph1*, to generate a BC_1_ population. The BC_1_ plants were then recurrently pollinated with Paragon *Ph1/Ph1* to produce BC_2_, BC_3_, BC_4_ and BC_5_ lines. 439 crossed seed were germinated and 278 plants were grown to maturity. Self-fertilization was facilitated by bagging three heads in each plant from the BC_2_ generation onward. Cross fertility was calculated as the number of crosses producing seed.

### Genotyping With KASP Markers

In this work, we used a set of previously produced 183 KASP markers polymorphic between hexaploid wheat and *Ae. caudata* accession 2090001 (Grewal et al., 2020a). Genomic DNA was isolated from leaf tissue of 10-day old seedlings in a 96-well plate as described by Thomson and Henry (1995).

Back-crossed and self-fertilized BC_x_F_y_ (where *x* = 1–5 and *y* = 0–2) lines were genotyped alongside five wheat genotypes (Chinese Spring, Paragon, Pavon, Highbury, and Alcedo) and the *Ae. caudata* accession as controls. Even though 278 plants were grown to maturity, only those plants in the BC_1_, BC_2_, and BC_3_ generations that produced either crossed or self-fertilized seed were genotyped. The wheat (Alcedo)-*Ae. caudata* B-G addition were also used as control lines to verify the specificity of the KASP markers (Grewal et al., 2020a) to each of the *Ae. caudata* chromosomes.

For each KASP^TM^ assay, two allele-specific primers and one common primer were used (Supplementary Table S1). Genotyping reactions were performed as described in Othmeni et al. (2019) using a ProFlex PCR system (Applied Biosystems by Life Technology). A final reaction volume of 5 μl was used which included 1 ng genomic DNA, 2.5 μl KASP reaction mix (ROX), 0.068 μl primer mix and 2.43 μl nuclease free water. PCR conditions were set as 15 min at 94°C; 10 touchdown cycles of 10 s at 94°C, 1 min at 65–57°C (dropping 0.8°C per cycle); and 35 cycles of 10 s at 94°C, 1 min at 57°C. QuantStudio 5 (Applied Biosystems) was used for fluorescence detection of the reactions and the data was analyzed using the QuantStudio^TM^ Design and Analysis Software V1.5.0 (Applied Biosystems).

### Multi-Color Genomic *in situ* Hybridization (mcGISH)

Preparation of the root-tip metaphase chromosome spreads, the protocol for mcGISH and the image capture was as described in Grewal et al. (2020a). In summary, genomic DNA from *Triticum urartu* (to detect the A-genome), *Aegilops speltoides* (to detect the B-genome), and *Aegilops tauschii* (to detect the D-genome) and *Ae. caudata* were isolated using extraction buffer [0.1M Tris-HCl (pH 7.5), 0.05M EDTA (pH 8.0), 1.25% SDS]. Samples were incubated at 65°C for 1 h before being placed on ice and mixed with ice cold 6 M NH_4_C_2_H_3_O for 15 min. The samples were then spun down, the supernatant mixed with isopropanol to pellet the DNA and the isolated DNA further purified with phenol/chloroform. The genomic DNA of (1) *T. urartu* was labeled by nick translation with ChromaTide^TM^ Alexa Fluor^TM^ 488-5-dUTP (Invitrogen; C11397; colored green), (2) *Ae. speltoides* was labeled by nick translation with DEAC-dUTP (Jena Bioscience; NU-803-DEAC; colored blueish purple), (3) *Ae. tauschii* was labeled with ChromaTide^TM^ Alexa Fluor^TM^ 594-5-dUTP (Invitrogen; C11400; colored red) and 4) *Ae. caudata* was labeled by nick translation with ChromaTide^TM^ Alexa Fluor^TM^ 546-14-dUTP (Invitrogen; C11401; colored yellow). Slides were probed using 150 ng of *T. urartu*, 150 ng of *Ae. speltoides*, 300 ng of *Ae. tauschii* and 50 ng of *Ae. caudata* labeled genomic DNAs, in the ratio 3:3:6:1 (green: blue: red: yellow). No blocking DNA was used. DAPI was used for counterstaining all slides. Metaphases were detected using a high-throughput, fully automated Zeiss Axio ImagerZ2 upright epifluorescence microscope (Carl Zeiss Ltd., Oberkochen, Germany) with filters for DAPI (blue), Alexa Fluor 488 (green), Alexa Fluor 594 (red), Alexa Fluor 546 (yellow) and DEAC (aqua). Image capture was performed using a MetaSystems Coolcube 1m CCD camera and image analysis was carried out using Metafer4 (automated metaphase image capture) and ISIS (image processing) software (Metasystems GmbH, Altlussheim, Germany).

### Fluorescence *in situ* Hybridization (FISH)

Root-tip metaphase chromosome spreads for Fluorescence *in situ* hybridization (FISH) were prepared as described above. Two repetitive DNA sequences, pSc119.2 (McIntyre et al., 1990), and pAs.1 (Rayburn and Gill, 1986) were used as probes. They were labeled with Alexa Fluor 488-5-dUTP (green) and Alexa Fluor 594-5-dUTP (red), respectively, and hybridized to the slide in the ratio 2:1 (green: red). Subsequent counterstaining and image capture were performed as described for GISH. The wheat FISH karyotype used for assigning chromosomes was that established by Tang et al. (2014).

## Results

### Assignment of KASP Markers to *Ae. caudata* Chromosomes

In a previous study, we reported the generation of 183 KASP markers polymorphic between hexaploid wheat and *Ae. caudata* accession 2090001 (Grewal et al., 2020a). Although, the physical location of these markers on the wheat genome was reported, the study did not address the physical location of these markers on the *Ae. caudata* genome.

In this work, the KASP markers were tested on the Alcedo-*Ae. caudata* addition lines carrying chromosomes B-G. It was their physical location and distribution on these C-genome chromosomes, among the addition lines, that determined the assignment of the *Ae. caudata* chromosomes to the wheat homoeologous groups. When a KASP marker detected the presence of *Ae. caudata* in any one of the addition lines, the marker was assigned to the corresponding C-genome chromosome. Of the 183 KASP markers tested, 156 were individually assigned to an *Ae. caudata* chromosome present in the disomic addition lines (Table 1). This set is missing an addition line for chromosome A, but reports have suggested it to be homoeologous to group 1 of wheat (Friebe et al., 1992; Danilova et al., 2017; Niu et al., 2018). Thus, of the remaining 27 markers, one marker failed and the other 26 markers that did not detect *Ae. caudata* in the addition lines B-G but amplified the *Ae. caudata* accession were assigned to chromosome 1C.

**TABLE 1:** Assignment of genome-specific and genome-non-specific KASP markers, and consequently homoeologous groups, to *Ae. caudata* chromosomes derived from Alcedo-*Ae. caudata* disomic addition lines and the distribution of the genome-specific KASP markers on the wheat A-, B-, and D-genomes.

*Ae. caudata* chromosome	Alcedo-*Ae. caudata* addition line	Genome-specific KASP markers			Genome-non-specific markers	Total number of KASP markers	Homoeologous group(s)
		A	B	D			
1C	–	7	7	9	3	26	1
2C	B	10	6	10	1	27	2/4
3C	F	2	6	7	7	22	3
4C	G	4	9	6	4	23	4/2/3/7
5C	C	8	14	10	1	33	5
6C	D	3	7	5	8	23	6
7C	E	6	4	15	3	28	7
Total		40	53	62	27	182	

There were 155 KASP markers specific to a wheat subgenome and 27 which had more than one corresponding sequence in the wheat genome and hence, were genome-non-specific for wheat (Supplementary Table S2). In total, 40 KASP markers were developed for the C-genome of *Ae. caudata* that were polymorphic with the A-genome of wheat, 53 with the B-genome and 62 with the D-genome of wheat. Chromosome 5C had the maximum number of KASP markers assigned to it (33) whereas the rest of the C-genome chromosomes had between 22 and 28 KASP markers assigned to them (Table 1). However, some C-genome chromosomes such as 2C and 4C, had markers assigned from non-homoeologous groups as shown in Table 1.

### Generation and Identification of Wheat-*Ae. caudata* Recombinant Lines

To generate introgressions from *Ae. caudata* into wheat, a crossing program was initiated between the two species that resulted in 165 F_1_ seeds from 49 crosses. A subsequent back-crossing program starting with 36 F_1_ plants and involving 439 back-crosses over five generations (F_1_-BC_4_) led to the production of 1,700 back-crossed seed and 3,496 self-fertilized seed. The number of seeds sown, germination rate, cross fertility and seed set, etc., are summarized in Table 2.

**TABLE 2:** Summary of number of seeds germinated and produced along with the number of crosses carried out and cross fertility in relation to the number of plants genotyped for each generation of the introgression program for *Ae. caudata* into wheat.

	Seeds sown	Plants grown to maturity	Germination rate (%)	Crosses made	Cross fertility (%)	Crossed seeds produced	Seeds/Cross	Self-fertilized seeds produced	Plants genotyped	Plants with *Ae. caudata* chromatin
Wheat × *Ae. Caudata*	–	–	–	49	43	165	3.4	–	–	–
F_1_	48	36	75	161	5	9	0.06	–	0	–
BC_1_	9	7	33	76	29	42	0.6	–	3	3
BC_2_	14	11	50	44	65	187	4.3	3	7	7
BC_3_	38	28	74	98	87	842	8.6	296	24	21
BC_3_F_1_	45	30	67	–	–	–	–	1217	30	15
BC_3_F_2_	18	11	61	–	–	–	–	*	11	7
BC_4_	81	50	62	60	100	620	10.3	1980	50	42
BC_4_F_1_	94	51	54	–	–	–	–	*	51	47
BC_5_	92	54	59	–	–	–	–	*	54	39
Total	439	278		488		1865		3496	230	181

*Plants are currently undergoing self-fertilization.

The KASP markers were also used to genotype a set of 230 wheat-*Ae. caudata* BC_x_F_y_ lines to detect the presence of *Ae. caudata* introgressions in wheat. Of the 230 plants genotyped, 181 plants were found to have *Ae. caudata* chromatin (recombinant chromosomes and/or whole chromosomes) which included 3 BC_1_, 7 BC_2_, 21 BC_3_, 15 BC_3_F_1_, 7 BC_3_F_2_, 42 BC_4_, 47 BC_4_F_1_, and 39 BC_5_ plants (Table 2). Analysis of the genotypes showed nine advanced back-crossed lines that had one or two recombinant chromosomes without the presence of any whole *Ae. caudata* chromosome (Table 3). Two recombinants were obtained from chromosome 1C, one from chromosome 4C, four from chromosome 5C and one from chromosome 7C. One introgression line had two of the above-mentioned recombinant chromosomes, one from 1C and one from 5C. Of the 7 BC_1_ plants grown to maturity, only 3 BC_1_ plants produced BC_2_ seed. All of the eight recombinant chromosomes were first observed in these 3 BC_1_ plants.

**TABLE 3:** List of wheat-*Ae. caudata* introgression lines containing recombinant chromosomes (RC) obtained in this study, the copy number of the RC(s) and the total number of chromosomes (2n) in each line.

Introgression line	Recombinant chromosome(s) (RC)	Copies of RC(s)	Total chromosome number 2n
BC_4_F_1_-157-9	T1AL-1CL.1CS	2	42
BC_4_F_1_-157-2	T5AL-5CL.5CS#1	2	42
BC_4_F_1_-158-12	T5AL-5CL.5CS#2	2	42
BC_4_F_1_-162-6	T5DL-5CL.5CS	2	42
BC_3_F_2_-160-4	T7DL-7CL.7CS	2	41
BC_4_F_1_-156-3	T1AL-1CL.1CS, T5AL-5CL.5CS#2	2	42
BC_4_-284-4	T1DS.1DL-1CL	1	42
BC_4_F_1_-164-2	T3DS.3DL-4CL	1	41
BC_3_F_1_-401-8	T5BL-5CL.5CS	1	42

**FIGURE 1:**
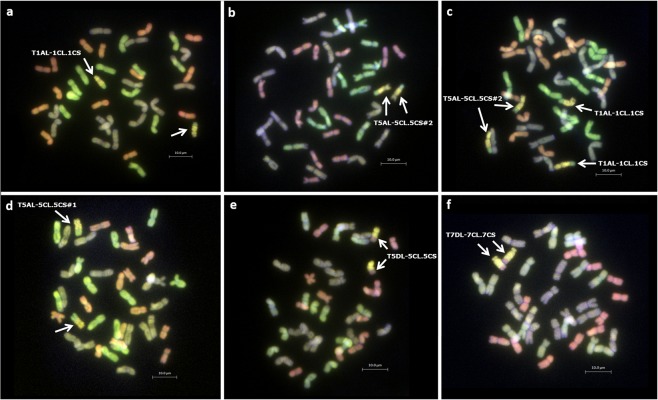
Identification of wheat-*Ae. caudata* recombinant chromosomes in homozygous lines through mcGISH analysis of root-tip metaphase chromosomes spreads. Arrows indicate recombinant chromosomes. **(a)** BC_4_F_1_-157-9, **(b)** BC_4_F_1_-158-12, **(c)** BC_4_F_1_-156-3, **(d)** BC_4_F_1_-157-2, **(e)** BC_4_F_1_-162-6, and **(f)** BC_3_F_2_-160-4. *Ae. caudata* segments (yellow) recombined with the A- (green) and D- (red) genome chromosomes of wheat. Wheat B-genome is represented as grayish/purple chromosomes.

**FIGURE 2:**
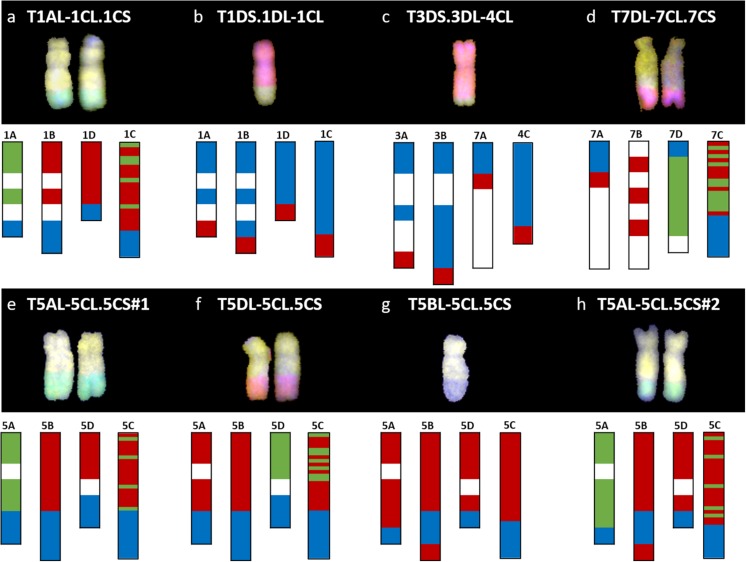
Molecular characterization of wheat-*Ae. caudata* introgression lines using KASP markers and mcGISH. Top: the mcGISH image of the recombinant chromosome(s) and bottom: the genotyping data with KASP markers of 8 wheat-*Ae. caudata* introgression lines. **(a)** BC_4_F_1_-157-9, **(b)** BC_4_-284-4, **(c)** BC_4_F_1_-164-2, **(d)** BC_3_F_2_-160-4, **(e)** BC_4_F_1_-157-2, **(f)** BC_4_F_1_-162-6, **(g)** BC3F1-401-8, and **(h)** BC_4_F_1_-158-12. C-genome segment is represented in yellow, A-genome in green, B-genome in blue, and D-genome in red. The genotyping is displayed with the markers distributed on the wheat chromosomes and the same set of markers ordered on the *Ae. caudata* chromosomes. White areas on the wheat chromosomes indicate the absence of KASP markers. Colored regions represent different genotype calls. Blue represents KASP markers homozygous for the wheat allele, red represents a heterozygous call and green represents a homozygous call for the *Ae. caudata* allele.

After self-fertilization, six of these BC_x_F_y_ lines showed stable homozygous introgressions which were validated by mcGISH analysis (Figures 1a–f). Five out of these six lines had 42 chromosomes with the sixth line having 41 chromosomes including the recombinant chromosomes (Table 3). However, prior to mcGISH analysis, the genome-specific KASP markers enabled the identification of the homozygous lines and the wheat chromosome that had recombined with the *Ae. caudata* segment in the homozygous recombinant lines (Figures 2a–h).

Figure 2 shows the mcGISH image of the recombinant chromosome(s) (top) and the KASP marker analysis (bottom) of eight wheat-*Ae. caudata* introgression lines. The genotyping is displayed with the markers physically located on the wheat chromosomes (for e.g., chromosomes 1A, 1B, and 1D in Figure 2a) and the same set of markers on the *Ae. caudata* chromosomes (for e.g., chromosome 1C in Figure 2a). When a line had a homozygous introgression, the KASP markers on the wheat chromosome involved in the recombination showed homozygous green calls as shown in Figures 2a,d,e,f,h. This is due to the genome-specificity of the KASP markers in wheat. Thus, when two copies of the *Ae. caudata* segment replaced both copies of the homoeologous wheat regions in the homozygous recombinant chromosomes, it resulted in no wheat allele for the genome-specific KASP markers in those regions. All other KASP markers detecting the *Ae. caudata* segment but not physically located on the recombinant chromosome show a heterozygous call to indicate the presence of *Ae. caudata* in a wheat background. The marker analysis in Figures 2a,e,h indicated the C-genome segment had recombined with the A-genome of wheat whereas in Figures 2b,f, the markers indicated the C-genome segment had recombined with the D-genome of wheat. The mcGISH analysis, above, confirmed these results.

When a line had a heterozygous introgression, as shown in Figures 2b,c,g, all the KASP markers that were located within the introgressed *Ae. caudata* segment gave a heterozygous call and thus, were not able to indicate which wheat chromosome the segment had recombined with. The mcGISH analysis (top), however, indicated the wheat genome, B (Figure 2g) or D (Figures 2b,c), present in these recombinant chromosomes.

### Physical Ordering of KASP Markers on C-Genome Chromosomes and Comparative Analysis With Wheat

**FIGURE 3:**
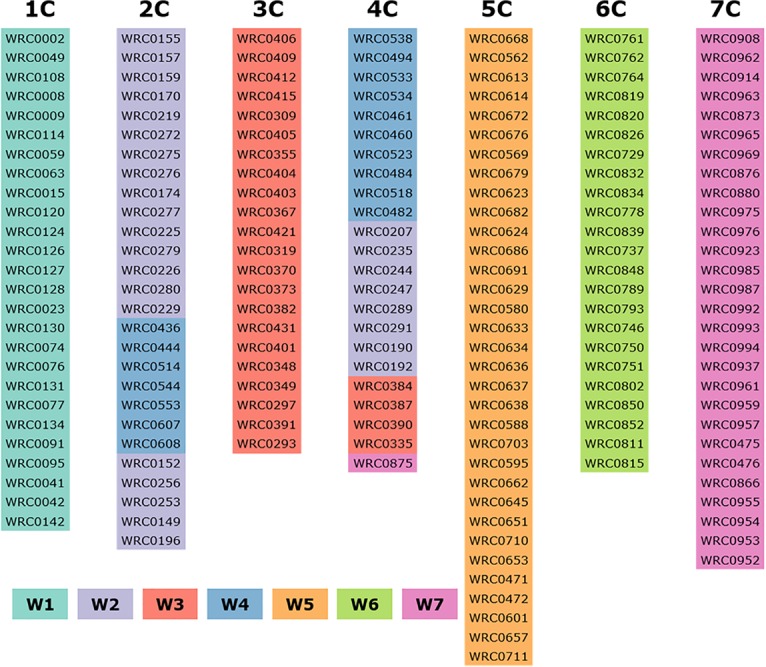
Physical order of the 182 KASP markers assigned specifically to each *Ae. caudata* chromosome. W means wheat and W1–W7 represents wheat homoeologous group 1–7, respectively. Chromosomes 2C showing markers from homoeologous group 2/4 and chromosome 4C showing markers from homoeologous groups 4/2/3/7.

**FIGURE 4:**
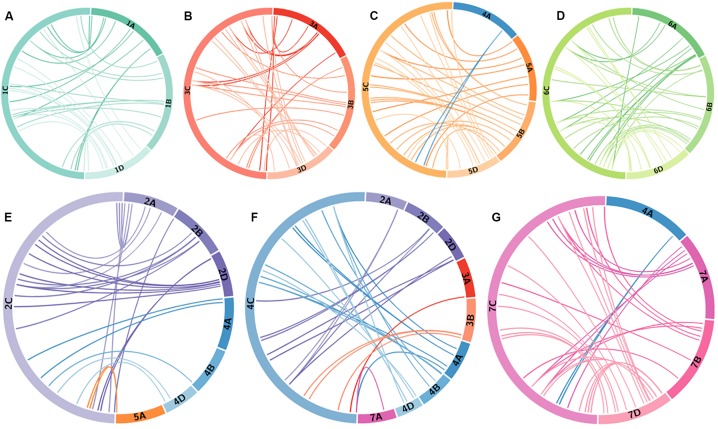
Comparative analysis of *Ae. caudata* chromosome rearrangement compared to wheat chromosomes revealed by physically mapped KASP markers. Circos diagrams of *Ae. caudata* chromosome **(A)** 1C, **(B)** 3C, **(C)** 5C, **(D)** 6C, **(E)** 2C, **(F)** 4C, and **(G)** 7C. Corresponding markers are linked between their tentative physical order on the *Ae. caudata* C-genome chromosomes and their physical location (Mb) on the orthologous regions on the wheat genome.

Based on the molecular marker analysis of the Alcedo-*Ae. caudata* addition lines (Table 1) and the wheat-*Ae. caudata* back-crossed population (Figure 2; bottom), the mcGISH analysis of the recombinant chromosomes (Figure 2; top) and previous reports on C-genome chromosomal rearrangements (Danilova et al., 2017), the 182 KASP makers were tentatively ordered onto the seven *Ae. caudata* C-genome chromosomes (Figure 3). A comparative analysis of the markers on the C-genome chromosomes and their physical positions on the wheat A, B, and D-genome chromosomes (Supplementary Table S2), as indicated by the wheat reference genome assembly RefSeqv1 (International Wheat Genome Sequencing Consortium [IWGSC]et al., 2018), is shown in Figure 4.

KASP marker analysis of the recombinant lines showed that markers WRC0002-0077 on chromosome 1C (Figure 3) corresponding to wheat homoeologous group 1 markers, spanning the short arm and approximately half of the proximal region of the long arm (Figure 4A), detected the *Ae. caudata* segment present in the recombinant chromosome T1AL-1CL.1CS. The spread of these markers across homoeologous group 1 also matched the size of the segment subsequently shown by mcGISH (Figure 2a). KASP markers WRC0095-WRC0142 on chromosome 1C (Figure 3), corresponding to the distal end of the long arm of homoeologous group 1 (Figure 4A), detected the *Ae. caudata* introgression present in T1DS.1DL-1CL subsequently validated by mcGISH (Figure 2b). This suggested that the marker sequences on chromosome 1C were potentially collinear with their counterparts on wheat chromosomes 1A, 1B, and 1D.

**FIGURE 5:**
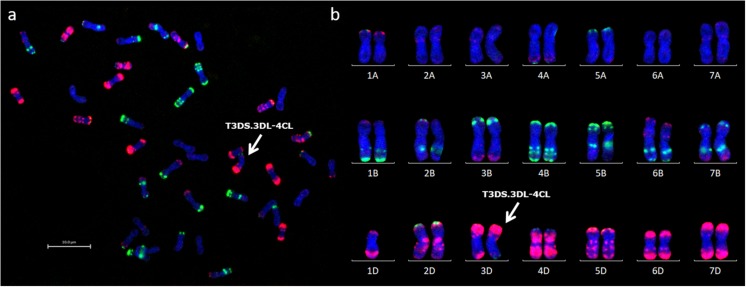
FISH analysis of wheat-*Ae. caudata* introgression line carrying recombinant chromosome T3DS. 3DL-4CL. **(a)** FISH image of a root-tip metaphase spread of chromosomes from line BC_4_F_1_-164-2. Arrow indicates recombinant chromosome T3DS. 3DL-4CL. **(b)** FISH karyogram of the same spread from line BC_4_F_1_-164-2 showing 41 chromosomes including one copy of recombinant chromosome T3DS. 3DL-4CL and one copy of chromosome 1D. Sites of hybridization are shown with fluorescence-labeled probes, pSc119.2 (green) and pAs.1 (red). Wheat chromosomes were assigned to the karyogram according to the FISH karyotype established by Tang et al. (2014).

Chromosome 4C of *Ae. caudata* had KASP markers from wheat homoeologous groups 2/3/4/7 assigned to it (Table 1). KASP markers WRC0387-0875 on chromosome 4C (Figure 3) detected the presence of an *Ae. caudata* segment in recombinant chromosome T3DS.3DL-4CL. However, Figure 4F shows that these markers were physically located on distal end of the long arm of wheat homoeologous group 3 (WRC0387, WRC0390 and WRC0335) with one marker near the centromere on homoeologous group 7 (WRC0875), indicating genomic rearrangements between homoeologous groups 3/4/7 in *Ae. caudata*. Due to the recombinant chromosome being present as a single copy in the introgression line, the KASP markers were not indicative of the wheat chromosome that had recombined with the *Ae. caudata* segment (Figure 2c). McGISH indicated it to be a D-genome chromosome which was subsequently confirmed to be chromosome 3D by FISH analysis (Figures 5a,b). The FISH analysis also showed that one copy of chromosome 1D was missing and hence, the total chromosome number for this line was 41 as mentioned in Table 3.

Four recombinants were obtained from chromosome 5C of *Ae. caudata*. KASP marker analysis showed that 21 markers, from WRC0668 to WRC0558 on chromosome 5C (Figure 3) detected all four recombinants. These markers correspond to wheat homoeologous group 5 spanning the small arm and approximately half of the proximal region of the long arm (Figure 4C). However, the four recombinants differed in their size and the wheat genome they had recombined with. For example, T5AL-5CL.5CS#1 and T5DL-5CL.5CS had the same 21 markers detecting the *Ae. caudata* segment but the former had recombined with chromosome 5A of wheat and the latter with chromosome 5D of wheat (Figures 2e,f, respectively). Recombinant chromosome T5BL-5CL.5CS had another 3 distal markers (WRC0703, WRC0595 and WRC0662; Figure 3), in addition to the 21, that detected the *Ae. caudata* segment in it and mcGISH analysis showed that it had recombined with the B-genome of wheat (Figure 2g). T5AL-5CL.5CS#2 had the longest segment from chromosome 5C of *Ae. caudata* and had another distal marker (WRC0645; Figure 3), making it a total of 25 markers, that detected this introgression. These markers indicated that recombination happened with chromosome 5A of wheat in this line and was also validated by mcGISH (Figure 2h). These segments assisted in the ordering of markers on chromosome 5C and comparative analysis showed that they were potentially collinear to corresponding sequences on wheat homoeologous group 5 (Figure 4C).

One large *Ae. caudata* segment was detected by 18 KASP markers, from WRC0908 to WRC0937 present on chromosome 7C (Figure 3). These markers indicated the segment to be to be homozygous and recombined with wheat chromosome 7D in the introgression line (Figure 2d). McGISH analysis of the recombinant chromosome T7DL-7CL.7CS showed that the introgressed *Ae. caudata* segment included the short arm and a large part of the proximal region of the long arm of chromosome 7C, with the distal end of the recombinant chromosome being replaced with the distal end of chromosome 7DL (Figure 2d). However, the markers found in this segment were found to be present on the proximal region of the short arm and the whole of the long arm of wheat homoeologous group 7 (Figure 4G). The markers corresponding to the short arm of wheat homoeologous group 7 were not detecting this recombinant chromosome and were thus, physically ordered onto the distal end of chromosome 7C (Figure 3) indicating an inversion in *Ae. caudata* group 7 compared to wheat.

Where markers could not be ordered due to absence of any recombination with wheat, such as for homoeologous group 2, 3, 4, and 6, the markers on chromosome 2C, 3C, 4C (except the distal end of the long arm) and 6C were ordered based on a previous study by Danilova et al. (2017).

## Discussion

### Assignment of KASP Markers to C-Genome Chromosomes Confirms Genomic Rearrangements

*Ae. caudata* has been shown to carry useful genetic diversity that can be used for wheat breeding. Its utilization for wheat improvement relies on the development of suitable and reliable molecular markers that can be used for the identification of *Ae. caudata* segments in introgression lines. In this research, a total of 182 KASP markers, developed in a previous study (Grewal et al., 2020a), were assigned to specific *Ae. caudata* chromosomes (Table 1), thereby, providing a rapid detection approach for the marker-assisted breeding of wheat-*Ae. caudata* introgression lines.

A number of studies have attempted assignment of various types of molecular markers to specific *Ae. caudata* chromosomes by testing them on the Alcedo-*Ae. caudata* disomic addition lines (Schubert and Blüthner, 1992, 1995). The variation observed in the results indicates the presence of genomic rearrangements. Recent studies using the Alcedo-*Ae. caudata* additions lines found that the C-genome chromosomes carried several inversions and translocations (Danilova et al., 2017; Gong et al., 2017; Niu et al., 2018). However, the deductions on the rearrangements carried by each chromosome were inconsistent among them. In this study, we used the same set of addition lines for the assignment of the KASP markers to the C-genome chromosomes and verified that *Ae. caudata* chromosomes B, C, D, E, F, and G were indeed 2C, 5C, 6C, 7C, 3C, and 4C respectively. Genotyping of these addition lines with the KASP markers showed that chromosome 2C had markers from wheat homoeologous groups 2/4 assigned to it (Table 1) which agreed with the work done by Danilova et al. (2017). Chromosome 4C had KASP markers assigned from homoeologous groups 4/2/3/7. This partially matched the results by Danilova et al. (2017) and Niu et al. (2018) who found that chromosome G (4C) had markers from homoeologous groups 4/2/3. Our studies found one marker from homoeologous group 7 assigned to chromosome 4C of *Ae. caudata*. Chromosome A is absent from this set of disomic addition lines. We assigned KASP markers that were not assigned to chromosomes B-G to chromosome A and found that they were all homoeologous to the group 1 chromosomes of wheat (Figure 4A) which is consistent with previous reports that used FISH with cDNA probes and determined that chromosome A of *Ae. caudata* is homoeologous to wheat group 1 chromosomes (Danilova et al., 2014, 2017).

### Generation of Wheat-*Ae. caudata* Recombinant Lines

It is to be noted that even though a considerable crossing program was undertaken, the eight wheat-*Ae. caudata* recombinant chromosomes were obtained from three BC_1_ parents. In the inter-specific F_1_ hybrids, which were haploid for the A, B, D, and C genomes, it is likely that the frequency of recombination between the chromosomes from different genomes would be low, in spite of the absence of the *Ph1* gene, leading to unviable gametes. This is exasperated by the genomic rearrangements in the C-genome possibly leading to inhibition of homoeologous chromosome pairing during meiosis. Hence, the cross fertility is lowest in the F_1_ hybrids at 5% but increases substantially in the back-cross generations reaching 100% in the BC_4_ population (Table 2). However, recombination events were first observed in the BC_1_ plants, having occurred in the F_1_ gametes, and no further homoeologous recombination was observed in subsequent back-cross generations.

### Genotyping With Genome-Specific KASP Markers Allows Identification of Homozygous Recombinant Lines and Their Physical Mapping on *Ae. caudata* Chromosomes

Genotyping of the back-crossed population with the KASP markers showed that the markers were not only able to detect *Ae. caudata* in the wheat background but also indicated whether a line had a homozygous segment and which wheat chromosome had recombined with *Ae. caudata* in a homozygous line. Eight out of nine introgression lines had a single type of recombinant chromosome (Table 3). The ninth line was homozygous for two of these recombinant chromosomes. As shown in Figure 2 (bottom), the KASP markers were able to detect whether a line was homozygous (red and green marker segments) or heterozygous (all red marker segments). A wheat chromosome that had green marker segments, representing homozygous *Ae. caudata* alleles, was shown to be involved in the recombinant chromosome. The genotyping results were also validated by mcGISH analysis of the recombinant lines (Figure 2; top).

Genotyping of these recombinant lines helped to physically order some of the KASP markers along the *Ae. caudata* chromosomes (Figure 3). For example, the recombinant line with chromosome 7C showed that markers homoeologous to the distal end of the short arm of wheat homoeologous group 7 were potentially present on the distal end of the long arm of chromosome 7C in *Ae. caudata* (Figure 4G). This agrees with a previous report by Danilova et al. (2017). Taking this into account and the other marker assignments discussed above, we concluded that our results are most concurrent with the conclusions by Danilova et al. (2017). Thus, for the regions of the C-genome where no recombination was obtained, the markers were ordered according to Danilova et al. (2017) (Figure 3); and compared to the wheat genome (Figure 4) which showed all the potential inversions and translocations found in the C-genome of *Ae. caudata*.

### Macrostructure of *Ae. caudata* Chromosomes

Chromosomes 1C, 5C, 6C, and 7C had maintained macro-synteny with wheat while chromosomes 1C and 5C were collinear with wheat and chromosomes 6C and 7C had inversions (Figure 4). In contrast, chromosomes 2C, 3C, and 4C had inter-chromosomal translocations and inversions (Figure 4). Due to the 4/5 translocation in wheat’s A-genome, Figure 4 showed markers corresponding to wheat chromosome 4A for *Ae. caudata* chromosome 5C (Figure 4C) and markers corresponding to wheat chromosome 5A for *Ae. caudata* chromosome 2C (that had a 2/4 translocation; Figure 4E). The macro-synteny and macro-collinearity with wheat could potentially be the reason we were able to obtain six recombinant chromosomes from *Ae. caudata* chromosomes 1C and 5C (Table 3 and Figure 2). Even though the majority of our results agreed with those of Danilova et al. (2017), we did not find any markers from wheat homoeologous group 7 translocated onto chromosome 6C (Table 1 and Figure 4F). Our work also found one marker from wheat homoeologous group 7 assigned to chromosome 4C (not found by Danilova et al., 2017) along with three markers from wheat homoeologous 3. This may be due to differences in the *Ae. caudata* accessions used in the two studies.

In contrast to most diploid and allopolyploid Triticeae, that have preserved chromosome macrostructure, rye (*Secale cereale*), *Aegilops umbellulata* and *Ae. caudata* genomes are highly rearranged (Devos et al., 1993; Zhang et al., 1998; Danilova et al., 2017). As a consequence of evolution, chromosome rearrangements in the genomes of wild relative species can disrupt the collinearity between the wild relative and the homoeologous wheat chromosomes (Devos et al., 1993; Zhang et al., 1998). This can result in reduction or absence of meiotic chromosome pairing. However, the recombinant chromosome, T3DS.3DL.4CL, along with a recombinant from chromosome 7C, T7DL-7CL.7CS (which has an inversion from the short arm onto the long arm compared to wheat) developed in this work show that it is possible to get wheat-wild relative recombination from wild relative chromosomes that are highly rearranged with respect to wheat.

Genomic rearrangements can be caused in Triticeae interspecific crosses due to gametocidal (Gc) chromosomes, also known as cuckoo chromosomes, which cause chromosome breakage, in the gametes that lack them (Endo, 1978; Finch et al., 1984; Nasuda et al., 1998; Friebe et al., 2000). Gc chromosomes have been found in many Aegilops species (Endo and Katayama, 1978; Endo, 1982, 2007) and in C-genome-derived allotetraploids where Gc action was found on chromosome 2C of *Ae. cylindrica* (Endo, 1996) and on chromosome 3C of *Ae. truncialis* (Endo and Tsunewaki, 1975). Gc action can range from lethal to semi-lethal depending on the wheat genotypes in which the Gc chromosomes are present (Endo, 1990). Some Gc chromosomes, such as 4S^sh^ from *Aegilops sharonensis*, can cause complete sterility in gametes that lack them in Chinese Spring wheat and are thus, preferentially transmitted to the offspring (Miller et al., 1982; Grewal et al., 2017). The Gc action of chromosome 2C is semi-lethal in Chinese Spring wheat, allowing minor structural rearrangements in the wheat chromosomes to be retained. However, chromosome 3C has a severe Gc action in Chinese Spring but mild or semi-lethal in other cultivars resulting in only slight chromosome damage. This suggests that there might be an inhibitor gene in those cultivars that prevents the Gc chromosomes from being preferentially transmitted (Endo and Gill, 1996). Our work does not show any preferential transmission of chromosome 2C or 3C in the back-crossed population (data not shown) suggesting a possible mild Gc action or the complete absence of it. In this work, Paragon wheat was used as the wheat background for the *Ae. caudata* introgressions. Thus, apart from the *Ph1* mutation in the F_1_ interspecific hybrids, a mild Gc action from C-genome chromosomes could potentially be responsible for the translocations between wheat and *Ae. caudata* chromosomes shown in this work. However, the results indicate that all recombinant chromosomes were obtained due to homoeologous recombination during meiosis. Six out of the eight recombinant chromosomes obtained in this work were between homoeologous linkage groups, i.e., chromosomes 1C of *Ae. caudata* with chromosomes 1A and 1D of wheat (Table 3) whereas any Gc action would have potentially resulted in random translocations. Confirmation of Gc action would need further cytological investigation of the gametophytes but it is also possible that the *Ae. caudata* accession used in this study does not possess any Gc action.

### Potential Benefits for Breeding Increased Disease Resistance

To exploit the potential of the C-genome, *Ae. caudata*-derived wheat introgression lines were previously developed and characterized, primarily for disease resistance genes. Dyck et al. (1990) found stem rust resistance in crosses between *Ae. caudata* and wheat monosomic 5B lines. Another study mapped *Ae. caudata*-derived leaf rust resistance on chromosome 2A of wheat (Iqbal et al., 2007) while two powdery mildew resistant quantitative trait loci (QTL) were mapped onto chromosomes 1A and 7A of wheat (Weidner et al., 2012). Riar et al. (2012) and Toor et al. (2016) mapped leaf rust and stripe rust resistance, respectively, onto chromosome 5DS of wheat in one wheat-*Ae. caudata* introgression line. Here, we report the generation of nine whea*t-Ae. caudata* introgression lines (Table 3), of which six had stable homozygous recombinant wheat-*Ae. caudata* chromosomes (Figure 1). These lines will be made available for further trait characterization studies.

Previous work investigating wheat-*Ae. caudata* introgression lines did not attribute the disease resistance QTLs they found in these lines to a specific *Ae. caudata* chromosome. Gong et al. (2017) and Niu et al. (2018) evaluated the Alcedo-*Ae. caudata* addition lines for agronomic traits such as resistance to rust and powdery mildew diseases. Addition lines D (6C), F (3C), and G (4C) showed some resistance to powdery mildew isolates (Niu et al., 2018) while addition line E (7C) showed a higher resistance to powdery mildew (Gong et al., 2017; Niu et al., 2018). Gong et al. (2017) suggested chromosome D (6C) carried leaf rust resistance while Niu et al. (2018), suggested that chromosome B (2C) exhibited resistance to leaf rust which was comparable to the *Ae. caudata* parent. While Gong et al. (2017) suggested that chromosome E (7C) might carry some stem rust resistance, Niu et al. (2018) found no stem rust resistance in the addition lines. In contrast, Xu et al. (2009) had shown that addition lines C (5C) and D (6C) were resistant to the Ug99 race group of the stem rust pathogen (*Puccinia graminis* f. sp. *tritici*). Consequently, a new gene for Ug99 resistance was introgressed from chromosome D (6C) into wheat (Xu et al., 2017). Investigations into grain quality traits showed that chromosome E (7C) or F (3C) could increase protein and wet gluten content when introduced into wheat (Gong et al., 2017). In this study, we have produced four wheat-*Ae. caudata* recombinant lines with wheat from chromosome C (5C) and one from chromosome E (7C) (Table 3), among others, which could potentially carry disease resistance and grain quality QTLs.

## Conclusion

Wheat-wild relative introgressions play an important role in wheat improvement and have been particularly exploited for disease resistance. However, a lack of compensation of the wheat genes by the wild relative chromosome segments and negative linkage drag can have a detrimental effect on agronomic performance of the wheat–wild relative introgression lines (Sears and Gustafson, 1993; Friebe et al., 1996). Therefore, better knowledge of the genome organization of the wild relative species is important before starting a new pre-breeding programs which can be laborious and costly. In this work, we produced KASP markers that proved to be valuable tools to detect *Ae. caudata* chromatin in a wheat background. In addition, these KASP markers have shed further light on the structural rearrangements present in *Ae. caudata*. We have also produced nine wheat-*Ae. caudata* recombinant lines of which six have stable homozygous introgressions that can be used for further trait analysis studies.

## Data Availability Statement

All datasets generated for this study are included in the article/Supplementary Material.

## Author Contributions

SG, MO, JW, CY, SH-E, DS, SA, IK, and JK carried out the crossing program. PI set up the high-throughput genotyping platform and developed the protocol. MO and JW performed the *in situ* hybridization experiments and the genotyping of wheat-*Ae. caudata* lines. SG analyzed the genotyping data, assigned the markers to the *Ae. caudata* chromosomes, and performed the comparative studies. SG, MO, IK, and JK conceived and designed the experiments. SG wrote the manuscript with assistance from MO, JK, and IK. All authors read and approved the final manuscript.

## Conflict of Interest

PI was employed by the company IDna Genetics Ltd. The remaining authors declare that the research was conducted in the absence of any commercial or financial relationships that could be construed as a potential conflict of interest.

## Supplementary Material

TABLE S1 Primer details for KASP assays.

TABLE S2 Physical location and allelic information of SNPs in the KASP assays.
